# Does Identity Incompatibility Lead to Disidentification? Internal Motivation to Be a Group Member Acts As Buffer for Sojourners from Independent Cultures, Whereas External Motivation Acts As Buffer for Sojourners from Interdependent Cultures

**DOI:** 10.3389/fpsyg.2017.00335

**Published:** 2017-03-07

**Authors:** Christina Matschke, Jennifer Fehr

**Affiliations:** ^1^Leibniz-Institut für WissensmedienTübingen, Germany; ^2^Hector Research Institute of Educational Science and Psychology, University of TübingenTübingen, Germany

**Keywords:** incompatibility, disidentification, self-construal, motivation, integration, acculturation

## Abstract

Most individuals possess more than one relevant social identity, but these social identities can be more or less incompatible. Research has demonstrated that incompatibility between an established social identity and a potential new social identity impedes the integration into the new group. We argue that incompatibility is a strong risk factor for disidentification, i.e., a negative self-defining relation to a relevant group. The current research investigates the impact of incompatibilities on disidentification in the acculturation context. We propose that incompatibility between one’s cultural identities increases the disidentification with the receiving society. It has, however, been shown that the motivation to be a group member serves as a buffer against negative integration experiences. Moreover, research from the intercultural domain has shown that intrinsic and extrinsic motivation has specific effects for members of cultures that differ in self-construal. In a European sample of High school exchange students (Study 1, *N* = 378), it was found that incompatibility was positively related to disidentification, but only for less (but not more) intrinsically motivated newcomers. In an Asian sample of international university students (Study 2, *N* = 74), it was found that incompatibility was also positively related to disidentification, but only for less (but not more) extrinsically motivated newcomers. Thus, the findings demonstrate that the effect of incompatibility between social identities on disidentification can be buffered by motivation. The results suggest that, depending on cultural self-construal, individuals have different resources to buffer the negative effect of incompatibility on the social identity.

## Introduction

Imagine Jane and Yukiko, two international sojourners who come to another country to study there. Jane is interested in the host culture and has always dreamt about living in that country. Yukiko’s parents have gone through great difficulties in order to offer Yukiko the best education abroad. Thus, both Jane and Yukiko are strongly motivated to become an integrated member of the receiving society. However, they soon encounter expectations of the receiving society that differ from what they are used to, based on their prior cultural identity. In other words, they experience incompatibility between their internalized primary cultural identity and the potential new social identity of the receiving society. One way to solve this conflict is to discard the new social identity. If social groups are omnipresent and one is confronted with the expectation to integrate, however, discarding an identity may be impossible. If the social group remains important for the self-concept but has a *negative* meaning, an individual might develop a disidentification with the group instead. Jane and Yukiko, for instance, may start to describe themselves as contrary to the people from the receiving society, to have negative feelings about these people and to act against the interests of the people. We argue that incompatibility between social identities increases the risk for a disidentification with a new group. It has, however, been shown that the motivation to be a group member can buffer incompatibility effects. The purpose of the current research is to investigate the impact of incompatibility on disidentification with a new social group, and the potential of motivation to serve as a buffer.

### Incompatibility Impedes Integration

Most individuals possess more than one relevant social identity. Social identities are, however, not always compatible. Newcomers of social groups seem to be especially sensitive to the relationship between standing social identities and the potential new social identity. It has been demonstrated that incompatibility between pre-existing social identities and the new group identity hampers the integration into new groups. Incompatibility has been conceptualized as cultural distance between primary and secondary culture (Babiker et al., 1980; Ward and Kennedy, 1992), inconsistent attitudes toward acculturation between the sojourner and the receiving society (Bourhis et al., 1997; Roccas et al., 2000; Zagefka and Brown, 2002), identity conflict (Leong and Ward, 2000; Ward et al., 2011) or psychologically felt incompatibility between identities (Benet-Martínez et al., 2002; Jetten et al., 2008; Cheng and Lee, 2009; Iyer et al., 2009; Martinovic and Verkuyten, 2012; Matschke and Fehr, 2015). The integration outcomes have been captured with measures of psychological and physical well-being (e.g., Babiker et al., 1980; Roccas et al., 2000; Chirkov et al., 2008), socio-cultural adaptation (Ward and Searle, 1990; Ward and Kennedy, 1992), intergroup relations (Zagefka and Brown, 2002), and social identification (Iyer et al., 2009; Matschke and Fehr, 2015). Despite these different conceptualizations and measurements, the core finding is that incompatibility makes the integration into a new group more difficult.

Research has, however, scarcely investigated the impact of incompatibility on *negative* integration outcomes. This is especially striking, as the societal discourse lies on negative outcomes, such as the separation of cultural subgroups, or acts of aggression and terrorism conducted by societal newcomers. Such passive and active harm toward one’s ingroup is often rooted in *disidentification* with that group (Elsbach and Bhattacharya, 2001; Becker and Tausch, 2014). Therefore, it is important to understand what factors affect newcomers’ disidentification with the receiving society, and what factors act as a buffer against disidentification. The current research is one of the few that investigates the impact of incompatibility on disidentification and the first to consider motivation as a buffer.

### Disidentification – The Outcome of Negative Integration Processes

For newcomers, the new group is a potential source of a new social identity. In the acculturation context, the receiving society is a relevant group and has a great potential to be included into the newcomers’ self-concepts, just like an ingroup (e.g., Berry, 1997; Sassenberg and Matschke, 2010). Whereas social identification is the positive form of such an inclusion, disidentification is the negative form. Disidentification is an active distancing of a group that is relevant for the self-concept. Disidentified group members feel contrary to the group and do not want to belong to that group (Elsbach and Bhattacharya, 2001; Becker and Tausch, 2014). Just like social identification (Tajfel and Turner, 1979), disidentification with a group affects an individual’s self-description, affect and behavior. Disidentified sojourners, for instance, would describe themselves as contrary to members of the receiving society, have a negative affective association to the group and act against the interests of the receiving society (Matschke and Sassenberg, 2010b). Social psychological research has created explicit multidimensional measures of disidentification that either focus on capturing the active distancing of an individual from a group (Matschke and Sassenberg, 2010b), or the feeling of opposition to the group behaviorally, cognitively and affectively (Becker and Tausch, 2014). Disidentification, captured with these explicit measures, has both specific antecedents and consequences. It is especially induced by negative experiences with a group, such as rejection (Matschke and Sassenberg, 2010b), a bad reputation (Elsbach and Bhattacharya, 2001), or illegitimate group assignment (De Vreeze and Matschke, 2016, unpublished). What makes the study of disidentification especially relevant is the finding that it predicts negative behavior toward the ingroup as well as other outgroups: disidentification is related to active and passive harm toward the ingroup (Becker and Tausch, 2014), to turnover (e.g., Elsbach and Bhattacharya, 2001; Matschke et al., 2016, unpublished), to anti-normative behavior (Matschke and Sassenberg, 2010a; Matschke et al., 2016, unpublished), to biased information behavior (De Vreeze and Matschke, 2016, unpublished; Matschke et al., 2016, unpublished), and to the derogation of other low status outgroups (Ikegami and Ishida, 2007; Matschke et al., 2016, unpublished). It is therefore important to reach a better understanding of the risk factors and buffers for disidentification in newcomers in order to increase our knowledge on negative integration outcomes.

Recent research has demonstrated that incompatibility between prior group memberships and new group memberships induces disidentification with the new group (De Vreeze et al., 2016, unpublished; Matschke et al., 2016, unpublished). In acculturation research, disidentification has mostly been captured indirectly with decreased levels of identification (e.g., Jasinskaja-Lahti et al., 2009), but recently direct measures of disidentification have successfully been used to investigate negative integration processes (Verkuyten and Yildiz, 2007; Matschke and Sassenberg, 2010b). The impact of incompatibility on disidentification has, however, not been investigated in the acculturation context. Based on the prior findings, we propose that incompatibility increases the risk for disidentification.

### Motivation to Be a Group Member

In the process of integrating oneself into a new group, feelings of incompatibility occur easily. Especially when living in a new cultural society, the experience of incompatibilities is rather the rule than the exception. But not all newcomers disidentify with their new groups. What resources have newcomers in coping with incompatibility that make them refrain from disidentification?

There is evidence that the motivation to be a group member plays a crucial role for the integration success. According to Self-determination theory (SDT; Deci and Ryan, 2000), there are different motivations to pursue one’s goals. Goals that fulfill the fundamental need for autonomy and self-determination are referred to as intrinsically motivated goals. Goals that are imposed upon individuals for reasons that lie outside their basic needs, like wishes of other persons or forces by circumstances, are referred to as extrinsically motivated goals. Based on this reasoning, research by Plant and Devine (1998) differentiates between intrinsic and extrinsic reasons for behavior. Whereas intrinsic motivation stems from internalized, personal beliefs, extrinsic motivation reflects the desire to avoid negative evaluations by others. Across different contexts, it has been shown that intrinsically motivated individuals are more successful in their goal pursuit than extrinsically motivated individuals (Plant and Devine, 1998; Deci and Ryan, 2000).

Recently, motivation has also become a matter of interest in the context of acculturation. Applied to the acculturation context, Chirkov et al. (2007, 2008) demonstrated that a self-determined motivation of students to study abroad increased their wish to acculturate, their socio-cultural adaptation, their subjective and physical well-being and the self-determination of academic activities. Thus, intrinsic motivation seems to increase the chances of successful acculturation processes. But why is intrinsic motivation so effective in goal pursuit?

According to the Self-completion theory (SCT; Wicklund and Gollwitzer, 1982) intrinsic motivation drives success because it leads to persistence in goal-pursuit. In other words: intrinsically motivated individuals are more successful because they do not give up when they meet obstacles. When intrinsically motivated goals are unfulfilled, a disagreeable sense of incompleteness is induced. In order to overcome this sense of incompleteness, more intrinsically motivated individuals increase their efforts to reach their goals. In contrast, goals with less intrinsic motivation do not induce this sense of incompleteness and will therefore be dropped when there are obstacles (Brunstein and Gollwitzer, 1996; Fehr and Sassenberg, 2009).

The superiority of intrinsic over extrinsic motivation is not, however, as global as assumed at first: There are theoretical accounts and empirical evidence that this finding is specific to certain cultures and may not be valid for individuals of all cultures (e.g., Markus and Kitayama, 1991; Iyengar and Lepper, 1999). We return to this question of the possible cultural specificity of intrinsic versus extrinsic motivation effects in the second study. Nevertheless, in Western cultures, it has been demonstrated that intrinsic motivation helps individuals to pursue their goals when they meet difficulties.

Sojourners that live in a foreign country with the goal to acculturate to the receiving society are likely to meet obstacles in form of identity incompatibility. Based on SDT and SCT, we suggest that intrinsic motivation buffers the effect of incompatibility on disidentification. In support of this idea, it has been shown that only less (but not more) intrinsically motivated newcomers disidentify with the new group when they feel rejected (Matschke and Sassenberg, 2010b). Moreover, Matschke and Fehr (2015) found that incompatibility impairs a developing *social identification* (i.e., a positive outcome criterion) of newcomers only if these are less (but not more) intrinsically motivated. We therefore hypothesize that incompatibility will be positively related to disidentification with the receiving society only for less (but not more) intrinsically motivated sojourners.

## Study 1

### Method

#### Design, Participants, and Procedure

A correlational study with the continuous independent variables *incompatibility* and *intrinsic* as well as *extrinsic motivation* and the criterion *disidentification* was conducted. A sample^1^ of 378 European exchange students (277 females, 97 males, 4 did not indicate gender, age *M* = 17, range 15–19 years) from 26 European countries, who had spent an exchange year in another European country, where they lived in local families and visited local High Schools filled in a questionnaire at their final seminar before returning home. Juristically, during the exchange year, i.e., also during the time when the data was collected, the organization stands in the position of the guardian of the adolescents. The organization encouraged the study and approved the questionnaire in detail. The questionnaire was voluntary, students were informed about the content of the study and consented to participate. There was, however, no written informed consent of the participant, because at the time of data collection, the approval of the guardian or organization was the ethical recommendation. Questionnaires were either in English (*N* = 184) or German (*N* = 193) and were introduced as a study about experiences during the exchange. Participants filled in measures on their intrinsic and extrinsic motivation to spend an exchange year in their host country, incompatibility, disidentification, and demographics. Debriefing was conducted via e-mail.

#### Measures

*Motivation* was measured with four items adapted from the Academic Self-Regulation-Questionnaire (Ryan and Connell, 1989). Two items measured intrinsic motivation (*r* = 0.33, *p* < 0.001, “I went as an exchange student to my host country because I felt like it” and “…because I thought that the experiences that I make in my host country would be fun,” *M* = 5.83, *SD* = 1.09), two items measured extrinsic motivation (*r* = 0.41, *p* < 0.001, “…because I thought that it would be appreciated by people that are important to me.” and “…because I wanted others to see that I can do it,” *M* = 3.36, *SD* = 1.65). Intrinsic and extrinsic motivation measures were unrelated, *r* = -0.06, *p* = 0.271.

*Incompatibility* was operationalized as divergence of acculturation strategies (Bourhis et al., 1997; see also Roccas et al., 2000, for a similar conceptualization). Primary culture incompatibility was captured by two measures: (1) participants’ attitudes toward the maintenance of the primary culture, captured with five items (α = 0.70, e.g., “It is important that exchange students maintain their home country’s way of living”) adapted by Zagefka and Brown (2002) and Geschke et al. (2010), and (2) the perceived attitudes of the host country, captured with the item “It is generally not popular in my host country if exchange students from my home country maintain their way of living.” Likewise, secondary culture incompatibility was captured by two measures: (1) the exchange students’ own attitudes on participation and contact with the secondary culture, captured with eight items (α = 0.78, e.g., “It is important that exchange students participate in their host country’s culture”) developed by ourselves and adapted from Zagefka and Brown (2002) and Geschke et al. (2010), and (2) the perceived attitudes of the host country, captured with the item “It is not liked in my host country when exchange students from my home country get into contact with local people.” In order to represent that only the combination of strong attitudes and strong meta-attitudes induce a psychologically meaningful incompatibility, the product^2^ of the attitudes and the meta-attitudes was calculated as an individual measure of incompatibility for each acculturation conflict. As a common measure of incompatibility, the two conflict measures (*r* = 0.34, *p* < 0.001) were aggregated (*M* = 14.66, *SD* = 7.60). The incompatibility measure was unrelated to intrinsic motivation (*r* = -0.03, *p* = 0.59), and positively related to extrinsic motivation (*r* = 0.21, *p* < 0.001).

Ten items measured *disidentification* (α = 0.88). The items were adapted from Matschke and Sassenberg (2010a). This scale has a strong focus on cognitive (e.g., “I make myself aware that there are other groups and countries besides my host country that are important to me), affective (e.g., “I feel bad when I meet people from my host country”) and behavioral (e.g., “I doubt that I will keep in contact with my host country for long”) distancing of an individual from a specific group.

All other items were assessed on a 7-point scale (1 = *I don’t agree at all*, 7 = *I fully agree*).

### Results

It was expected that incompatibility is positively related to disidentification, but only for those who are less (but not more) in intrinsic motivation. In order to test for potential effects of demographic variables, the independent variables and the criterion were correlated with gender and age. The correlation with gender indicated than females experiences less incompatibility (*r* = 0.16, *p* = 0.002) and disidentification (*r* = 0.19, *p* < 0.001), but were higher in intrinsic motivation (*r* = -11, *p* = 0.025), all other correlations remained non-significant, all *r*s < 0.09, all *p*s > 0.12. We therefore control for gender and age in the following analyses.

In order to test whether the buffering effect is specific to intrinsic motivation, a hierarchical regression analysis with the control variables gender and age and the continuous predictors Incompatibility, Intrinsic motivation, and Extrinsic motivation was conducted on disidentification. In the first step, gender and age were entered in the analysis. In the second step, the main effects were entered in the analysis, then the predicted Incompatibility × Internal motivation interaction (step 3), followed by the Incompatibility × External motivation interaction (step 4). Note that we did not expect or test the Incompatibility × Intrinsic motivation × Extrinsic motivation interaction. The regression analysis (see **Table 1**) revealed in the first step, that gender was indeed related to disidentification throughout the models, whereas the relation between age and disidentification remained marginal and became non-significant when other variables were entered into the model. In the second step, the analysis revealed that stronger Incompatibility was related to stronger disidentification, *B* = 0.025, *SE* = 0.004, *p* < 0.001, whereas Intrinsic motivation, *B* = -0.03, *SE* = 0.04 *p* = 0.421, and Extrinsic motivation *B* = 0.03, *SE* = 0.02, *p* = 0.204, were unrelated to disidentification. When entered into the model separately, the expected Incompatibility × Intrinsic motivation was significant, *B* = -0.11, *SE* = 0.04; *p* = 0.003, and remained significant with *B* = -0.12, *SE* = 0.04, *p* = 0.002, when the not significant Incompatibility × Extrinsic motivation, *B* = -0.05, *SE* = 0.04, *p* = 0.217, was entered into the model.

**Table 1:** Coefficients of the hierarchical regression in Study 1 (*N* = 378).

	1	2	3	4
Gender	0.15^∗∗^	0.11^∗^	0.09^∗^	0.10^∗^
Age	0.09^+^	0.08	0.07	0.07
Incompatibility		0.25^∗∗∗^	0.27^∗∗∗^	0.27^∗∗∗^
Intrinsic motivation		-0.04	-0.04	-0.04
Extrinsic motivation		0.06	0.05	0.06
Incompatibility × Intrinsic motivation			-0.15^∗∗^	-0.16^∗∗^
Incompatibility × Extrinsic motivation				-0.06


*^+^*p* < 0.10, ^∗^*p* < 0.05, ^∗∗^*p* < 0.01, ^∗∗∗^*p* < 0.001.*

**FIGURE 1:**
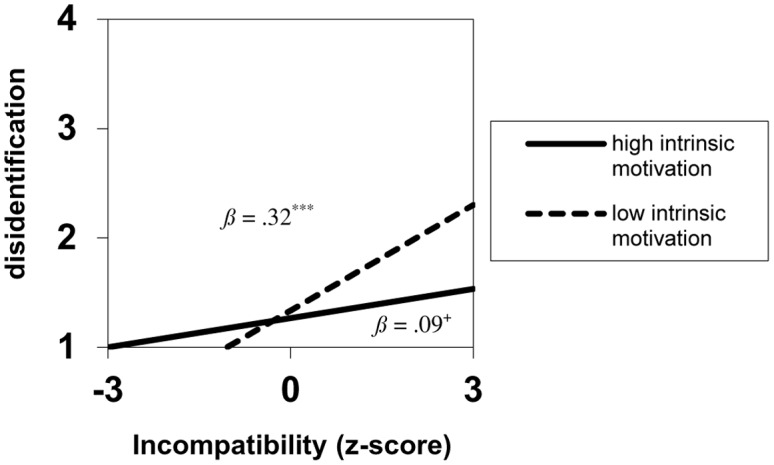
**Disidentification as a function of acculturation incompatibility for individuals with independent self-construal in Study 1 (*N* = 378)**.

Simple slope analyses resolved the Incompatibility × Intrinsic motivation interaction (Aiken and West, 1991, with standardized independent variables). The analyses revealed that when less intrinsically motivated (1 *SD* below the mean), Incompatibility was positively related to disidentification, *B* = 0.32, *SE* = 0.05, *p* < 0.001. When more intrinsically motivated (1 *SD* above the mean), Incompatibility was less strongly related to disidentification, *B* = 0.09, *SE* = 0.06; *p* = 0.079 (see **Figure 1**).

### Discussion

Study 1 investigated a sample of European sojourners. It was expected and found that incompatibilities between acculturation attitudes are positively related to disidentification with the receiving society, but only if sojourners are less intrinsically motivated. When more intrinsically motivated, this relation did not disappear completely, but sojourners were less strongly affected by incompatibility. Extrinsic motivation did not affect disidentification, nor did it buffer the incompatibility effects. Study 1 thus supports the prediction that intrinsic motivation has the power to buffer the integration process from difficulties: it weakens the negative effects of incompatibility on disidentification.

#### Intercultural Differences in Motivation Effects

Study 1 demonstrated that intrinsic (but not extrinsic) motivation specifically buffered the effects of incompatibility on disidentification. There is, however, reason to suppose that even extrinsic motivation may serve as a buffer against incompatibility, depending on the self-construal of individuals in certain cultures. Across a wide range of theories, it is suggested that individuals differ across cultures in whether they construe their self as individualistic or independent vs. collectivistic or interdependent (e.g., Hofstede, 1980; Triandis, 1989; Markus and Kitayama, 1991; Singelis and Brown, 1995). Individuals with independent self-construal’s experience and behavior are guided by their own inner thoughts, emotions, and actions. Individuals with interdependent self-construal’s experience and behavior are guided by the thoughts, emotions, and actions of relevant others.

There is reason to suppose that individuals with interdependent self-construal are strongly driven by extrinsic motivation, because they are more strongly motivated by the goals of relevant others (Markus and Wurf, 1987)^3^. They are more motivated by activities that significant others chose for them, and more willing to be guided by significant others’ opinions than individuals with independent self-construal (Markus and Kitayama, 1991; Cialdini et al., 1999; Hernandez and Iyengar, 2001; Iyengar and Brockner, 2001; Kitayama and Uchida, 2005). Motivation and persistence are stronger for individuals with interdependent self-construal, if the task is extrinsically motivated, compared to individuals with independent self-construal, whose motivation and persistence are strongest when intrinsically motivated (Iyengar and Lepper, 1999; Heine et al., 2001). Individuals with interdependent self-construal also prefer choices for others over choices for themselves, whereas the pattern is the opposite for individuals with independent self-construal (Pöhlmann et al., 2007). Study 1 demonstrated that intrinsic, but not extrinsic motivation buffered the relation between incompatibility and disidentification. Study 1, however, only tested this effect in Europeans, who are considered to be independent in self-construal (Hofstede, 1980; Markus and Kitayama, 1991). Based on the findings on motivation and self-construal, we suggest that for individuals from cultures with interdependent self-construal, extrinsic motivation is a buffer against the relation between incompatibility and disidentification. More precisely, we predict in a sample from a culture of interdependent self-construal that for less (but not more) extrinsically motivated newcomers, incompatibility is positively related to disidentification.

## Study 2

Study 2 is a correlational study with a small sample of individuals from different Asian countries. Asian cultures are considered to be cultures with interdependent self-construal (Hofstede, 1980; Markus and Kitayama, 1991). The study aims at collecting first evidence for the prediction that for individuals with interdependent self-construal, extrinsic motivation serves as a buffer against the effect that incompatibility has on disidentification.

### Method

#### Design, Participants, and Procedure

Study 2 was a correlational study that investigated the continuous independent variables incompatibility, intrinsic, and extrinsic motivation and the criterion disidentification in a sample of individuals from cultures that are interdependent in self-construal. Seventy-four^4^ Asian students from 10 different countries (with the majority of 54 from Malaysia, 68 females, 6 males, age *M* = 22.58, range 19–48 years), who had moved to New Zealand about *Median* = 5 months (range: 0.5–156 months) ago in order to study at a local university, participated in the study. The questionnaire included an information sheet that informed the participants that their completion of the questionnaire will be understood as consent for the use and publication of the data. Study 2 was approved by the ethical committee of the School of Psychology, Victoria University Wellington (New Zealand). Participants filled in English language measures on intrinsic and extrinsic motivation to move to New Zealand, disidentification, and demographics, as well as other measures irrelevant to the research question. The study was part of a larger data collection advertised in mailing lists and introduced at meetings for international students as a study about experiences in New Zealand that could be filled in online (*N* = 4) or paper-and-pencil (*N* = 70). Participants were debriefed via e-mail.

#### Measures

As the motivation measure of Study 1 was rather short, in Study 2 motivation was measured with an adapted version of the Self-Regulation-Questionnaire – Study abroad (short version; Chirkov et al., 2008). The questionnaire captures intrinsic motivation to move to New Zealand with four items including intrinsic and identified regulation (α = 0.81; e.g., “I moved to New Zealand because I thought it was an exciting thing to do,” *M* = 3.43, *SD* = 1.06). Extrinsic motivation was measured with six items that captured both extrinsic and introjected motivation (α = 0.80, e.g., “I moved to New Zealand because others (relatives and friends) forced me to do this,” *M* = 2.04, *SD* = 0.95). Note that all extrinsic motivation items describe reasons to study abroad that are connected to one’s relationships to others. The items were assessed on a 5-point scale (1 = *not at all because of this reason*, 5 = *completely because of this reason*). Intrinsic and extrinsic motivation measures were correlated, *r* = 0.46, *p* < 0.001.

*Incompatibility* (α = 0.76; *M* = 2.02, *SD* = 0.63) was measured with seven items adapted from the cultural distance scale of Babiker et al. (1980) and Piontkowski et al. (2000). Perceived differences between culture, religion, climate, food, educational level, material comfort, family life, and people in general were rated on a 5-point scale (1 = *I don’t agree at all*, 5 = *I fully agree*). Incompatibility was unrelated to intrinsic and extrinsic motivation, both *r* < |0.15|, *p* > 0.20.

*Disidentification* (α = 0.68, *M* = 2.29, *SD* = 0.73) was measured as in Study 1, but one item (“I tell myself that I have other groups where I can play a part in”) was deleted from the scale to improve intrinsic consistency (α = 0.71). Thus, the final scale consisted of nine items.

### Results

It was expected that for individuals with interdependent self-construal, incompatibility is positively related to disidentification, but only for less (but not more) extrinsically motivated sojourners. In order to test for potential effects of demographic variables, the independent variables and the criterion were again correlated with gender and age. Age was related to incompatibility, *r* = -0.27, *p* = 0.019, all other correlations remained non-significant, all *r*s < 0.17, all *p*s > 0.14. As in Study 1, we control for gender and age in the following analyses. The main prediction was tested in a hierarchical regression analysis with the control variables gender and age, and the continuous predictors Incompatibility, Extrinsic motivation, and Intrinsic motivation on disidentification. Demographics were entered into the analysis first, then the main effects (step 2), the Incompatibility × External motivation interaction (step 3), followed by the Incompatibility × Internal motivation interaction (step 4). For the demographic variables, the analysis (see **Table 2**) revealed no significant effects of gender and age, all βs < 0.18, all *p*s > 0.14. In step 2, neither the main effects of Incompatibility, *B* = 0.04, *SE* = 14; *p* = 0.789, nor Intrinsic motivation *B* = -0.04, *SE* = 0.09, *p* = 0.684, were significant. Extrinsic motivation was positively related to disidentification, *B* = 0.24, *SE* = 0.10, *p* = 0.023. When entered into the model separately, the expected Incompatibility × Extrinsic motivation was significant, *B* = -0.28, *SE* = 0.10, *p* = 0.006. When the not significant Incompatibility × Internal motivation, *B* = -0.20, *SE* = 0.15, *p* = 0.191, was entered into the model, the Incompatibility × External motivation interaction remained marginally significant, *B* = -0.21, *SE* = 0.11, *p* = 0.070.

**Table 2:** Coefficients of the hierarchical regression in Study 2 (*N* = 74).

	1	2	3	4
Gender	-0.06	-0.15	-0.15	-0.18
Age	-0.10	-0.05	0.02	0.02
Incompatibility		0.03	0.20	0.18
Extrinsic motivation		0.32^∗^	0.11	0.17
Intrinsic motivation		-0.05	-0.14	-0.34^+^
Incompatibility × Extrinsic motivation			-0.42^∗∗^	-0.31^+^
Incompatibility × Intrinsic motivation				-0.28


*^+^*p* < 0.10, ^∗^*p* < 0.05, ^∗∗^*p* < 0.01, ^∗∗∗^*p* < 0.001.*

**FIGURE 2:**
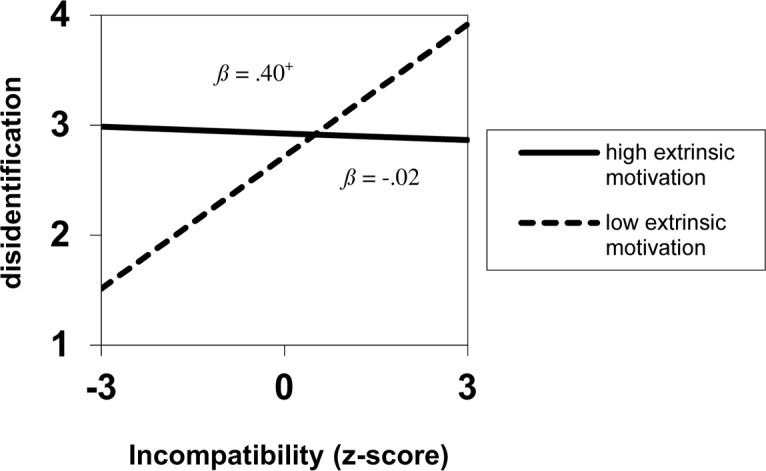
**Disidentification as a function of Cultural distance for individuals with interdependent self-construal in Study 2 (*N* = 74)**.

When the Incompatibility × Extrinsic motivation interaction was resolved into simple slopes (Aiken and West, 1991, with extrinsic motivation standardized, 1 *SD* above/below the mean), the predicted pattern was found: incompatibility was marginally related to disidentification only for less extrinsically motivated sojourners, *B* = 0.40, *SE* = 0.21, *p* = 0.066, whereas more extrinsically motivated sojourners’ disidentification was unaffected by incompatibility, *B* = -0.02, *SE* = 0.14, *p* = 0.887 (see **Figure 2**).

### Discussion

Study 2 investigated the effect of incompatibility and motivation on disidentification in a sample from a culture with interdependent self-construal. It was expected that incompatibility is only positively related to disidentification for those lower (but not higher) in extrinsic motivation. The data provided first evidence that extrinsic motivation has the predicted buffering effect on the relation between incompatibility and disidentification. This interpretation can, however, only be made with caution, because the effects of Study 2 may be underpowered, and Study 2 can only be taken as first evidence which should stimulate future research.

Intrinsic motivation did not significantly affect the relation between incompatibility and disidentification in Study 2, but there was a descriptive relation between the two constructs that might reach conventional levels of significance in studies with greater power. Moreover, both types of motivation were, other than in Study 1, correlated. The fact that extrinsic, but not intrinsic motivation served as a buffer in Study 2 strengthens the assumption that extrinsic motivation is a stronger resource in a sample from interdependent cultures compared to independent cultures, but it cannot be ruled out that any kind of motivation, or both intrinsic and extrinsic motivation serve as buffers for incompatibility experiences in samples from cultures with interdependent self-construal.

## General Discussion

Two correlational studies investigated the effect of incompatibility and motivation on disidentification with the new group in the acculturation context. It was expected that motivation buffers the effect of incompatibility on disidentification with the new group. The data supported the predictions. In a European sample (Study 1), incompatibility was positively related to disidentification, but only if newcomers were less (not more) intrinsically motivated. In an Asian sample incompatibility was also positively related to disidentification, but only when newcomers were less (not more) extrinsically motivated.

### The Impact of Incompatibility

When individuals possess several group memberships, it is easily possible that these are incompatible and difficult to reconcile in the self-concept (Roccas and Brewer, 2002; Amiot et al., 2007). Individuals who move to another country to live there are likely to experience incompatibility somehow or other. Research has demonstrated that incompatibility hampers the integration process (e.g., Jetten et al., 2008; Iyer et al., 2009; Matschke et al., 2016, unpublished). The current research used two different kinds of incompatibilities that sojourners may experience: differences in acculturation attitudes and cultural distance. Whereas differences in acculturation attitudes are a rather subjective measure, cultural distance is more objective. Both measures do not directly capture whether individuals actually think that the differences are problematic (compare, for instance, Matschke and Fehr, 2015). Despite these differences, the patterns are similar, and both types of incompatibilities could be buffered by motivation. The ability to keep on track despite cultural or attitudinal differences between oneself and the new group may prove essential for the healthy integration process. Even though incompatibilities have received considerable attention by acculturation researchers, motivation has so far never be considered as a buffer for incompatibility experiences, let alone in a way that differentiates between extrinsic and intrinsic motivation (for an exception, see Matschke and Fehr, 2015). The current research contributes to the growing body of evidence that demonstrates that motivation can make a difference in the integration process, especially when the process turns out to be thorny.

### Disidentification in Integration Processes

The present research is one of the few that take a deeper look into negative integration outcomes. When newcomers are strongly immersed in their group, as is the case on sojourns and during migration, it is unlikely that the self-concepts of newcomers remain untouched by incompatibility. Most research that investigated the impact of incompatibility of multiple social identities has captured positive outcomes, such as the lack of social identification or socio-cultural adaptation (e.g., Ward and Kennedy, 1992; Iyer et al., 2009; Matschke and Fehr, 2015). So far, negative outcomes of incompatibility captured by research were passive constructs like the indifference toward the receiving society (e.g., anomie or individualism in Bourhis et al.’s model) and physical symptoms (e.g., headaches or other health issues; Chirkov et al., 2008; for an exception, see Verkuyten and Yildiz, 2007; Matschke and Sassenberg, 2010a,b). Thus, individuals with incompatible multiple identities are often treated as passive victims of the negative integration processes. By investigating disidentification, which is an important predictor of negative behavior toward an ingroup (Becker and Tausch, 2014), group members are considered an active part of a negative integration process. Acts of aggression conducted by newcomers underline the importance of the investigation of active, negative integration outcomes in newcomers. Even if acts of aggression are, of course, the tip of the iceberg, disidentification in group newcomers is hardly constructive for the long-term integration success. It is therefore important to investigate the early development of disidentification in newcomers.

### Motivation as a Resource

Motivation seems to be a powerful resource when people enter new groups: it can harden people’s persistence toward difficulties. Even though different motives to move abroad have been suggested some 20 years ago (e.g., Bierbrauer and Pedersen, 1996; Berry, 1997; Ward et al., 2001), the differentiation into different kinds of motivation is still under-investigated. In line with SDT and SCT, Study 1 demonstrated in a European sample that especially intrinsic motivation hardens people’s persistence toward difficulties. However, Study 2 suggests that in Asian samples, extrinsic motivation may play a similar buffering role as internal motivation does in the European sample. This finding supports the assumption that individuals with different self-construal are motivated by different things. Individuals with interdependent self-construal rely more strongly on the judgment of significant others and take their opinions more strongly as a guideline for their own behavior than individuals with independent self-construal (see also Hernandez and Iyengar, 2001). The present research thus underlines that the principles of SDT and SCT are applicable to more than one cultural background, if adjusted to the context: it is not only the intrinsic motivation that leads to success (Deci and Ryan, 2000) or persistence (Wicklund and Gollwitzer, 1982), but individuals with interdependent self-construal can similarly be driven by extrinsic motivation. Theories on motivation should take these differences more strongly into account and acknowledge that there may be different paths to a healthy goal-pursuit.

### Limitations and Future Avenues

Studies 1 and 2 contain two correlational datasets that differ in a variety of dimensions (e.g., age, intercultural context, host culture, temporary vs. permanent residence) in addition to self-construal. Thus, the interpretation of the results as a cross-cultural comparison can only be made with caution. In fact, self-construal has not been measured in the current set of studies, but was derived from the cultural background of the sample. This approach, however, over-simplifies individuals, as they differ in their self-construal also within cultures (Markus and Kitayama, 1991). Future research should replicate the present findings in one sample with heterogeneous self-construal and use it as a factor in the design, e.g., by measuring self-construal in an intercultural sample of sojourners or by manipulating self-construal (e.g., Kühnen et al., 2001) in order to rule out alternative explanations for the differences in the samples.

The differences in the relations between intrinsic and extrinsic motivation between the two samples is another indication that the samples differ in their motivational structure. Considering that studying abroad is something that usually only high social class background people can afford, and that high social class background is related to independent (rather than interdependent) self-construal (e.g., Stephens et al., 2012), the specific pattern of intrinsic and extrinsic motivation in Study 2 might also be due to a high social class sample from Asia. Thus, the sample might not be purely interdependent in their self-construal. Measuring self-construal and social class background in future studies could deepen our understanding of different sources of self-construal and their effects on motivation.

The finding that intrinsic motivation is a strong resource for individuals with independent self-construal seems to be robust, whereas the evidence whether individuals with interdependent self-construal are driven by extrinsic or intrinsic motivation is somewhat mixed. On the one hand, Chirkov et al. (2007, 2008) found, for instance, that the effects of self-determined motivation on socio-cultural adaptation are not significant in the Chinese sample compared to the international sample. On the other hand, the relation between the self-determined motivation and well-being was found, but weaker for Asians than for Europeans. Research on the persuasiveness of messages has demonstrated that collectivistic advertisements are more appealing in collectivistic societies than in individualistic societies, but that under certain circumstances, members from collectivistic cultures find both individualistic and collectivistic messages appealing (Han and Shavitt, 1994). Taken together, there is evidence that intrinsic motivation might be more universal in its effect than assumed, and that only extrinsic motivation is especially effective in interdependent cultures. Thus, individuals with interdependent self-construal might benefit from both intrinsic and extrinsic motivation to be a group member. In other words, any kind of motivation would be useful when these newcomers experience incompatibility. In the context of international students, it is likely that the students were, one way or the other, motivated to live in the receiving society. There are, however, certainly contexts (e.g., individuals who take refuge from war, natural disasters, political persecution or poverty) where the motivation to come live in another country is less driven by the receiving society itself, but by a motive to leave the country of origin. The present research only indirectly captures the effects of amotivation and does not explicitly investigate the effects of low levels of both intrinsic and extrinsic motivation. It demonstrates, however, that it is worthwhile to investigate the effects of different kinds of motivations, and future research should broaden our knowledge about the relation between kinds of motivation (including amotivation), self-construal, incompatibility, and integration outcomes.

### Practical Implications

The current findings demonstrate that incompatibility is a risk factor for disidentification. Disidentification has disagreeable and potentially dangerous consequences for both the new group and the individual trajectories within the group. These findings have practical implications for the avoidance of undesirable negative integration processes. One way to minimize the risk of disidentification is to decrease incompatibility. This could be done by negotiating differences in acculturation attitudes, by encouragement of perspective taking, or by a focus on similarities rather than differences (compare Rosenthal and Crisp, 2006). Experiences of incompatibility are, however, likely to occur in the intercultural context and sometimes impossible to rule out (e.g., cultural distance). In these cases, the motivation of newcomers should be strengthened. The current research suggests that it is worthwhile to focus on both intrinsic and extrinsic motivation, because both kinds of motivation may help newcomers not to be discouraged when they experience incompatibilities. Thus, interviewers in selection processes should ask both “Why do you want to be a part of the group?” as well as “What does your social context think about you’re joining the group?”. When individuals with interdependent self-construal express that they have been “sent” by, for instance, parents, Westerners should stop seeing these people as pitiful, hard-driven individuals that are not allowed to follow their nature, but consider that for individuals with interdependent self-construal, the goals and opinions of relevant others are just as motivating and gratifying as inner needs and wishes are for individuals with independent self-construal. For the training of newcomers, it seems promising to strengthen both kinds of motivation, for instance by having them recall their inner reasons for the group membership or think about significant others that are happy about their being in the group.

### Conclusion

The current study is the first to demonstrate the joint impact of incompatibility between different social identities and motivation in different cultures on the newcomers’ social identities. It thus contributes to research on multiple identities as well as motivation and cross-cultural research. The experience of incompatibilities between pre-existing and new social identities is a normal and probably unavoidable part of an integration process. The current research demonstrates that individuals have resources to stay on track and refrain from disidentification despite these difficulties. These resources are, however, not the same for everyone. It is thus a fruitful research avenue to investigate what resources are effective in whom to improve the integration processes when social identities turn out to be incompatible.

## Author Contributions

CM lead the conception and design of the work, the data collection, conducted the analyses and lead the interpretation of the data, and drafted the manuscript. JF contributed substantially to the analyses and the interpretation of the data, and revised the manuscript critically. CM and JF approve the final version of the manuscript and agree to be accountable for all aspects of the work in ensuring that questions related to the accuracy or integrity of any part of the work are appropriately investigated and resolved.

## Conflict of Interest Statement

The authors declare that the research was conducted in the absence of any commercial or financial relationships that could be construed as a potential conflict of interest.

## Footnotes

1 *Post hoc* linear multiple regression, fixed model, *R*^2^ increase, power analysis conducted with G^∗^Power 3.1.5 (Faul et al., 2007), parameters set to effect size *R*^2^ change = 0.029, alpha = 0.05, sample size *N* = 378, one tested predictor, five total predictors, indicated a good power of 0.95.

2 This measure is similar to the discrepancy measure introduced by Roccas et al. (2000). We chose to use the product of participants’ own attitudes and the meta-attitudes because of the negative framing of the meta-attitudes. Moreover, the product reflects that only larger differences between sojourners’ and receiving societies’ attitudes results in psychologically felt incompatibilities, whereas lower passion toward attitudes, or lower meta-attitudes, should not result in a psychologically relevant feeling of conflict.

3 While Markus and Kitayama (1991; see also Iyengar and Lepper, 1999) argue that individuals with interdependent self-construal have motivation driven by others that would become intrinsic, in classic terms of SDT this kind of motivation would be called *extrinsic* motivation, i.e., the motivation to do things enforced by circumstances or *relevant other persons*. For the sake of differentiation while reading this paper, we will continue to refer to the construct as *extrinsic* motivation.

4 *Post hoc* linear multiple regression, fixed model, *R*^2^ increase, power analysis, parameters set to effect size *R*^2^ change = 0.095, alpha = 0.05, sample size *N* = 74, one tested predictor, seven total predictors, indicated a medium power of 0.84. A sample size of *N* = 92 would have had a good power of 0.90 (*A priori* linear multiple regression, fixed model, *R*^2^ change with parameters set as described as above).
